# Genome-Wide Identification, Characterization and Expression Analysis of *Lipoxygenase* Gene Family in *Artemisia annua* L.

**DOI:** 10.3390/plants11050655

**Published:** 2022-02-28

**Authors:** Ying Meng, Yu Liang, Baosheng Liao, Wenrui He, Qianwen Liu, Xiaofeng Shen, Jiang Xu, Shilin Chen

**Affiliations:** 1 College of Pharmacy, Shandong University of Traditional Chinese Medicine, Jinan 250000, China; mengying3374@sina.com; 2 Institute of Chinese Materia Medica, China Academy of Chinese Medical Sciences, Beijing 100000, China; liangyu94@126.com (Y.L.); swjs082lbs@126.com (B.L.); l349295748@126.com (Q.L.); xfshen1030@163.com (X.S.); 3 College of Pharmaceutical Science, Dali University, Dali 671000, China; wenrui96017@163.com; 4 School of Pharmacy, Chengdu University of Traditional Chinese Medicine, Chengdu 610000, China

**Keywords:** lipoxygenase, *Artemisia annua* L., gene family, bioinformatics, expression pattern

## Abstract

Lipoxygenase (LOX) is a ubiquitous oxygenase found in animals and plants and plays a pivotal role in diverse biological processes, including defense and development. Artemisinin, which can only be obtained from *Artemisia annua* L., is the most effective therapeutic drug for malaria without serious side effects. This study identified and analyzed *LOX* gene family members in the *A. annua* genome at the chromosomal level. Twenty *LOX* genes with various molecular weights, isoelectric points, and amino acid numbers were identified and named *AaLOX*, which were located in the cytoplasm or chloroplast. The average protein length of all *AaLOX* was 850 aa. Phylogenetic tree analysis revealed that the *AaLOX* was divided into two major groups, 9-LOX and 13-LOX. The exon numbers ranged from 1 to 12, indicating that different *AaLOX* genes have different functions. The secondary structure was mainly composed of alpha helix and random coil, and the tertiary structure was similar for most *AaLOX*. Upstream promoter region analysis revealed that a large number of cis-acting elements were closely related to plant growth and development, light response, hormone, and other stress responses. Transcriptome data analysis of different tissues suggested that the gene family was differently expressed in the roots, stems, leaves, and flowers of two *A. annua* strains HAN1 and LQ9. qRT-PCR confirmed that *AaLOX5* and *AaLOX17* had the highest expression in flowers and leaves. This study provides a theoretical basis for the further functional analysis of the *AaLOX* gene family.

## 1. Introduction

Lipoxygenases (linoleate:oxygen oxidoreductase, EC 1.13.11; LOXs) represent a kind of heme dioxygenase that contains iron ions, specifically catalyzes the oxygenation of polyunsaturated fatty acids, and is widely expressed in animals, plants, and fungi. As a key rate-limiting enzyme in plant fatty acid metabolism pathway (LOX pathway), LOX specifically catalyzes the oxygenation of polyunsaturated fatty acids with cis and cis-1 cis-4-pentadiene structure to produce two kinds of fatty acid hydroperoxide (HPOs): 13-HPOT and 9-HPOT [1,2]. In particular, 13-HPOT is catalyzed by 13-LOX and is a necessary step in the jasmonic acid (JA) pathway [3]. As an important signal molecule in plants, JA participates in growth and development and mediates the defense response of plants against herbivores and microbial pathogens [4]. Another metabolite produced are green leaf volatiles (GLVs), which are involved in plant defense responses and have direct or indirect inhibitory effects on pathogens and pests by inducing the expression of related defense response genes [5].

Genes encoding *LOX* have been identified in many plant species, including six *LOX* genes in *Arabidopsis thaliana*, 14 *LOX* genes in rice [6], eight *LOX* genes in *pepper* [7], 18 *LOX* genes (five pseudogenes) in *Vitis vinifera* [8], and 21 *LOX* genes in *Populus trichocarpa* [9]. *LOX* has multiple physiological functions in plants and is mainly involved in physiological processes, such as seed metabolism, fruit ripening [10], and leaf senescence [11]. This enzyme also plays an essential role in biotic and abiotic stresses, such as external damage [12], pest feeding [13], and pathogen infection [14,15,16]. In *A. thaliana*, *AtLOX1* is involved in the defense response of pathogens in leaves [17], and *AtLOX2* is involved in jasmonic acid (JA) biosynthesis [18]. *AtLOX3* and *AtLOX4* play important roles in flower development and male fertility regulation [19], and *AtLOX5* is involved in plant lateral root development and defense response [20]. *AtLOX6* is expressed in roots and participates in JA synthesis [21]. The study of *LOX* gene family in *A. annua* is of great importance to its growth and defense function. 

The dry, aboveground part of *A. annua* is used as a traditional Chinese medicine and has a medicinal history of more than 2000 years in China. Sesquiterpenes are the main medicinal active components of *A. annua*, among which, artemisinin is the main ingredient used in the treatment of malaria, which is included in the International Pharmacopoeia published by the World Health Organization [22]. Youyou Tu, who discovered artemisinin, won the 2015 Nobel Prize in Physiology or Medicine with William C. Campbell and Satoshi Omura for their work on new treatments for parasitic diseases. *A. annua* also has other pharmacological effects, such as anti-virus [23], antifungal and bacteriostatic [24], anti-inflammation [25], anticancer [26], and immunomodulatory [27]. Therefore, increasing the artemisinin content and improving the stress resistance of *A. annua* in complex environment are of great importance. 

The chromosome level genome of *A. annua* has been assembled and uploaded to Global Pharmacopoeia Genome Database (http://www.gpgenome.com, accessed on 20 August 2021). However, the *LOX* genes of *A. annua* have not been reported. In this study, the *LOX* genes of *A. annua* were studied at the whole genome level. Subsequently, 20 *LOX* genes were identified and analyzed by bioinformatics to provide a reference for exploring the growth, development, and stress resistance of this plant.

## 2. Results

### 2.1. Identification and Analysis of the Basic Physicochemical Properties of AaLOX Family Members in A. annua

Twenty-six candidate *LOX* protein sequences were identified from *A. annua* genome. After false positive and incomplete sequences were removed, 20 *AaLOX* protein sequences were obtained as follow-up analysis objects (Table S1), named *AaLOX1*-*AaLOX20*. The predicted proteins contained only one LOX domain [20,28] or one PLAT/LH2 domain [29]. The number of *AaLOX* genes in *A. annua* is more than that in *A. thaliana*, cucumber [30], grape [31], and apple [32].

Analysis of the physical and chemical properties of these proteins showed that their length ranged from 130aa (*AaLOX20*) to 1217 aa (*AaLOX10*) and most were longer than 850 aa. The corresponding molecular weight ranged from 15093.37 Da(*AaLOX20*) to 139132.95 Da(*AaLOX10*). The theoretical isoelectric point varied from 5.22 (*AaLOX19*) to 6.89 (*AaLOX15*). The instability indexes of seven *LOX* family members, such as *AaLOX12*, *AaLOX8* and *AaLOX11* were all less than 40, indicating their stability. The other proteins with instability coefficient more than 40 were unstable. The mean values of grand average of hydropathicity (GRAVY) were negative, indicating that the *AaLOX* proteins were hydrophilic. According to subcellular localization prediction, nine proteins were located only in chloroplast, three were located only in cytoplasm, and eight were located in chloroplast and cytoplasm.

### 2.2. Chromosomal Distribution and Collinearity of AaLOX Genes

**Figure 1:**
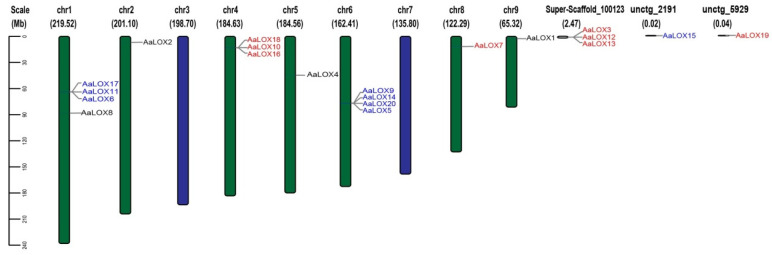
Distribution of *AaLOX* genes on *A. annua*. Chromosome names are shown on top of the chromosome and gene names are shown on the right side of the chromosome. According to the phylogenetic tree, the closely related genes are shown in the same color. The chromosomes are indicated by different colors, green for the presence of *AaLOX* and blue for the absence of *AaLOX*. The scale bar on the left indicats the length (Mb) of *A. annua* chromosomes.

On the basis of the chromosome distribution of 20 *AaLOX* genes in *A. annua*, the *AaLOX* family was unevenly distributed on the eight chromosomes and scaffold of *A. annua*. Among them, four *AaLOX* genes were located on chr1 and chr6, one *AaLOX* gene on chr2, chr5, chr8, and chr9, three *AaLOX* genes on chr4, and no *AaLOX* gene was found on chr3 and chr7. Other *AaLOX* genes were located in scaffold or contig (Figure 1).

### 2.3. Selective Pressure Analysis

The evolutionary selection pressure of *AaLOX* family members was analyzed (Table S2). Among the six homologous gene pairs, only one *AaLOX* pair had a Ka/Ks ratio larger than 0.5 (but less than 1). The remaining Ka/Ks ratios were less than 0.5, suggesting that most of the *AaLOX* genes underwent strong purifying selection.

### 2.4. Phylogenetic Analysis of AaLOX Genes

**Figure 2:**
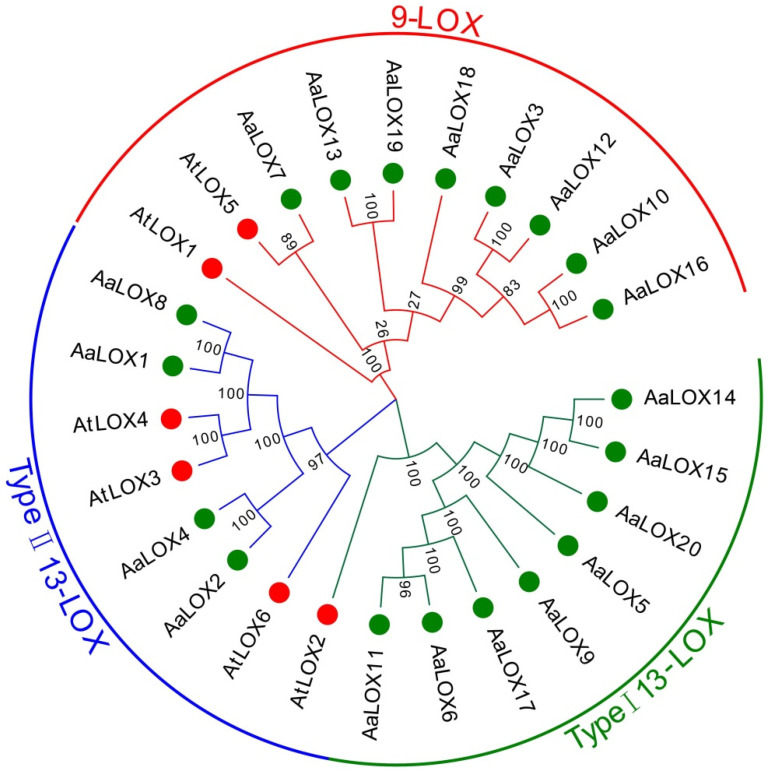
Phylogenetic analysis of *LOX* genes from *A. annua* and *A. thaliana*. Gene classes are indicated by different colors. Taxon labels are depicted in red for the 9-LOX clade, in blue for type II 13-LOX clade, and in green for type I 13-LOX clade. Members of *LOX* protein from *A. annua* are denoted by a green dot.

All the *LOX* family members were highly conserved in evolution. According to classification of *A. thaliana*, the *AaLOX* genes family can be accurately divided into three groups: 9-LOX subfamily, type Ⅰ 13-LOX, and type Ⅱ 13-LOX (Figure 2).

Eight type Ⅰ 13-LOX and four type Ⅱ 13-LOX were identified. *AaLOX1* and *AaLOX8* in type Ⅱ 13-LOX were closely linked to *AtLOX3* and *AtLOX4*. *AtLOX4* plays an important role in *A. thaliana* seed development and can defend against nematode infection. Eight 9-LOX members were found. Among them, *AaLOX7* was closely connected to *AtLOX5*, which is involved in lateral root development and defense response to pathogens in *A. thaliana*, suggesting that *AaLOX7* has similar functions in *A. annua* [33,34].

### 2.5. Exon–Intron Gene Structure and Conserved Motif Analysis

**Figure 3:**
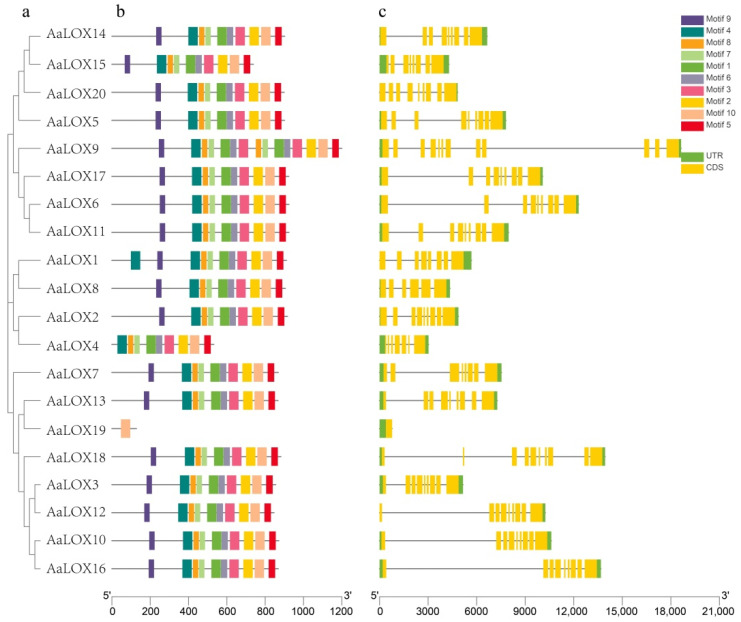
Gene structure and conserved motif of *AaLOX* genes. (**a**) Phylogenetic tree. Neighbor-joining (NJ) tree was constructed using MEGA X. (**b**) Conserved motif. The colored boxes on the right denote 10 motifs. (**c**) Gene structure. The yellow boxes, black lines, and green boxes represent exon, intron, and untranslated region (UTR), respectively.

A NJ phylogenetic tree was constructed using the 20 protein sequences of *AaLOX* (Figure 3a), and 10 conserved motifs were identified using the online software MEME (Figure 3b). Most of the *AaLOX* protein sequences had similar motif compositions and positionings, in the order of motifs 9, 4, 8, 7, 1, 6, 3, 2, 10, and 5. *AaLOX1* contained 11 motifs, with an extra motif 4. *AaLOX4* contained nine motifs but lacked motif 9. *AaLOX19* contained only one motif 10, and the rest of the *AaLOX* protein contained motifs 1–10.

Analysis on the exon-intron structure of the *AaLOX* protein sequences of *A. annua* (Figure 3c) may provide insights into the evolution of the *AaLOX* gene family. The results showed that most of the gene structures (number, length, and position of exons) of the members of the same subfamily were similar, indicating that the protein structure was conserved within the subgroup, but was highly specific among different subfamilies. The exon number of 20 *AaLOX* genes ranged from 1 to 12 and the number of introns was between 0 and 11. *AaLOX19* had no intron, which is similar to the *PtLOX5* of poplar [7]. A total of 168 exons and 148 introns were identified, implying the structural diversity of *AaLOX* genes. Compared with that of type Ⅰ 13-LOX and 9-LOX, the length of type Ⅱ 13-LOX genes was significantly shorter mainly reflected in their shorter and fewer introns. Gene structure and conserved motif arrangement, together with the phylogenetic analysis results, could strongly support the reliability of the classification.

### 2.6. Modeling of the Secondary and Tertiary Structural Homology of AaLOX Gene Family

The secondary structure of *AaLOX* gene family was analyzed (Table S3). The structural proportion from high to low was alpha helix, random coil, extended strand, and beta turn. Beta sheet was not included in all *AaLOX* genes. Alpha helix and random coil accounted for more than 80%, and extended strand and beta turn accounted for around 20%. Therefore, alpha helix and random states may play a major role in the secondary structure of *AaLOX* proteins.

**Figure 4:**
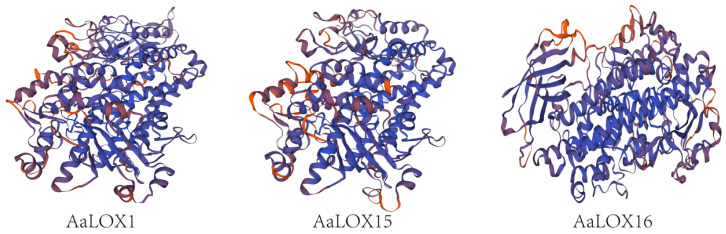
3D structure of *AaLOX* protein sequences.

*AaLOX1*, *AaLOX15,* and *AaLOX16* proteins as representatives of three *AaLOX* subfamilies were selected for tertiary structural homology modeling. The tertiary structure of other 17 *AaLOX* proteins are shown in Figure S1. Through blast comparison, 3PZW.1.A with the highest homology was selected as the best template for modeling and visualization (Figure 4). The tertiary structures of *AaLOX* proteins were similar, indicating that their high conservation.

### 2.7. Promoter Analysis of AaLOX Genes

**Figure 5:**
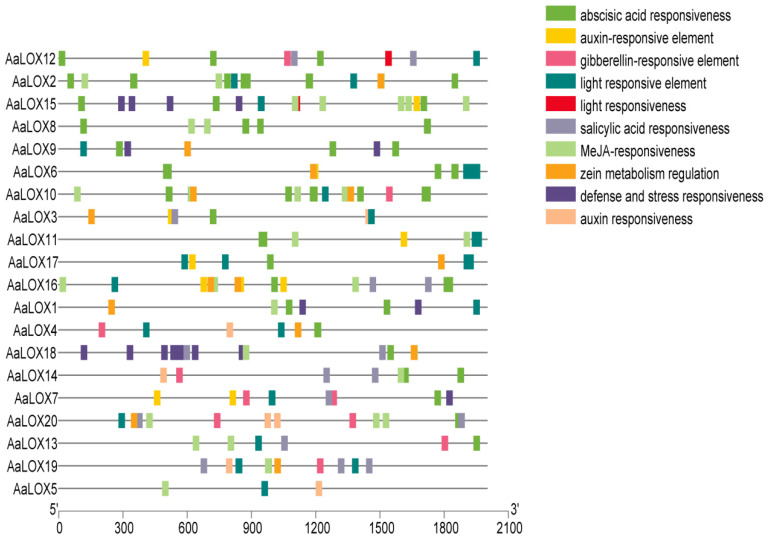
Prediction of cis-acting elements in the promoter of *AaLOX* gene family. The colored boxes on the right denote cis-acting elements and the same function are shown in the same color.

Cis elements distribution reflects the potential function and regulation of genes. Through the analysis of promoter cis-acting elements (Figure 5), the cis-acting elements related to auxin, salicylic acid, abscisic acid, methyl jasmonate, and gibberellin were enriched in the promoter region.

These cis-acting elements indicated that hormones have a potential regulatory effect on *AaLOX*. Each *AaLOX* member contains a different number of hormone response elements. Among which, *AaLOX15* contained the most cis regulatory elements related to stress response (15), followed by *AaLOX10* (13), *AaLOX16* (13), *AaLOX2* (12), *AaLOX18* (12), and *AaLOX20* (12).

In the *AaLOX* family, the cis regulatory element with strongest relation to stress response was ABA response elements (41), followed by methyl jasmonate (MeJA) response elements (28), light response elements (22), salicylic acid response elements (16), corn protein metabolism regulation and defense and stress responsiveness (14), auxin-responsive element (11), gibberellin-responsive element (10), and auxin responsiveness (six). The cis regulatory element with the weakest relation to stress response was light responsiveness (2). These comprehensive findings suggested that *AaLOX* can participate in a variety of responses.

In addition, the promoter regions of evolutionarily closely related *AaLOX* members usually contained similar cis regulatory elements. Except for *AaLOX4*, the promoter region of type Ⅰ 13-LOX subfamily contained light response elements. All type Ⅱ 13-LOX subfamily members contained ABA response elements, and most 9-LOX subfamily members contained salicylic acid and MeJA response elements.

### 2.8. GO Enrichment Analysis of AaLOX

**Figure 6:**
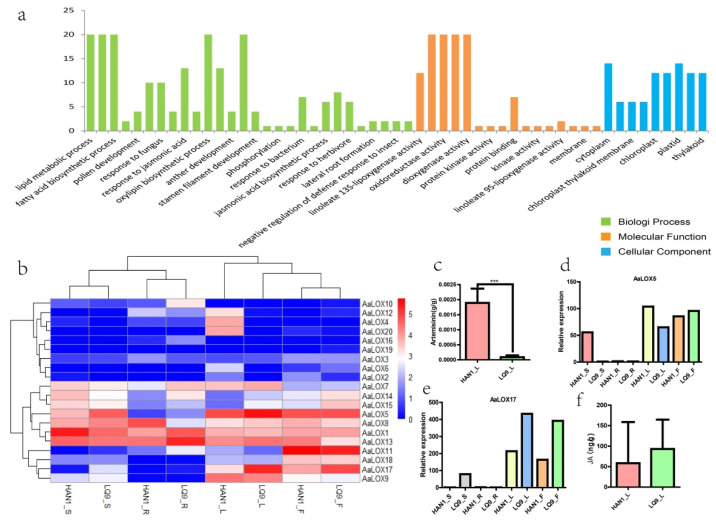
GO enrichment, expression profiling of *AaLOX* and JA and artemisinin content. (**a**) GO enrichment analysis of *AaLOX*, *** *p* < 0.001. (**b**) Expression of *AaLOX* genes in different tissues from the two strains. HAN1_R, HAN1_S, HAN1_L and HAN1_F represent the root, stem, leaf and flower of HAN1 respectively; LQ9_R, LQ9_S, LQ9_L and LQ9_F represent the root, stem, leaf and flower of LQ9, respectively. (**c**) Artemisinin content of two strains of *A. annua*. (**d**,**e**) represented the relative expression levels of *AaLOX5* and *AaLOX17*. (**f**) JA content of the two strains of *A. annua*.

Gene ontology (GO) functional enrichment analysis was carried out to further understand the function of *AaLOX*. The GO terms of *AaLOX* were divided into three categories: biological process, molecular function, and cellular component (Figure 6a). The largest number of GO entries was attributed to biological process (28 terms), followed by molecular function and cellular component. The biological processes were mainly oxylipin biosynthesis, oxidation-reduction, fatty acid biosynthesis, and fatty acid metabolism, which conformed to the function of *LOX*. The molecular functions mainly included oxidoreductase activity, metal ion binding, and dioxygenase activity. These results confirmed the functions of *AaLOX* in many biological processes, related to plant growth and development as well as biotic and abiotic stresses.

### 2.9. Tissue Specificity of AaLOX Gene Expression and JA and Artemisinin Content

The transcriptomic data of the roots, stems, leaves, and flowers of two strains of *A. annua* strains (HAN1 and LQ9) were analyzed to further study the expression pattern of *AaLOX* (Figure 6b). HAN1 was a high-artemisinin strain, and LQ9 was a low-artemisinin strain (Figure 6c). In HAN1, two, two, and three highly expressed genes were found in the stems, leaves, and flowers, respectively. In LQ9, four, three, and three highly expressed genes were observed in the stems, leaves, and flowers, respectively.

Bell [27] proved that *AtLOX2* is involved in JA synthesis, and the present NJ-tree indicated that *AaLOX9*, *AaLOX17*, *AaLOX5,* and *AtLOX2* were type Ⅰ 13-LOX genes. Subcellular localization results showed that these three genes were also located in chloroplast, similar to *AtLOX2*. Expression pattern analysis revealed that *AaLOX5* and *AaLOX9* were highly expressed in HAN1_L, and *AaLOX17* and *AaLOX5* were highly expressed in LQ9_L and LQ9_F, respectively. For pattern verification, the expression levels of two genes (*AaLOX5* and *AaLOX17*) in HAN1 and LQ9 tissues were analyzed by qRT-PCR (Figure 6d,e). The results showed that *AaLOX5* and *AaLOX17* genes were highly expressed in the leaves and flowers of HAN1 and LQ9, respectively, indicating that *AaLOX* expression varies in different tissues of HAN1 and LQ9 strains.

*LOX* is a key enzyme in the JA synthesis pathway. Hence, the JA content of HAN_L and LQ9_L was measured by UPLC-MS (Figure 6f). The results showed that the JA content differed between the two strains. In addition, JA is associated with artemisinin synthesis [32]. Significant differences in artemisinin content were found between the two strains, but the relationship among *AaLOX*, JA content and artemisinin level is still unknown. Further studies are needed to prove the relationship of *AaLOX* with JA content and artemisinin content.

## 3. Discussion

*A. annua* is an important traditional Chinese medicine, and its artemisinin component is the most effective drug against malaria [35]. This plant is the first internationally recognized natural medicine discovered in China and plays an essential role in human health [36]. *LOXs* have attracted great attention as key rate-limiting enzyme in plant fatty acid metabolism pathway. *LOX*-derived signaling compounds can be used in a wide range of applications, such as treatment of inflammation, cancer, food storage, and pest control [37].

In this study, a comprehensive analysis was conducted on the *LOX* genes of *A. annua*, including phylogenetic relationship, gene structure, domain structure, chromosomal location, selection pressure and expression pattern. The diversity of gene structures has aided the evolution of numerous gene families [38]. *AaLOX* members of the same subgroup had similar intron and exon quantities, indicating that *AaLOX* genes have a highly conserved structure, this finding was consistent with the results for *A. thaliana* and grape *LOX* genes [6,8]. Fourteen *AaLOX* genes had more than eight introns, and *AaLOX9* was the longest gene with 11 introns. Conservative motif analysis revealed that most *AaLOX* motifs were similar. It is suggested that these conserved motifs may play an important role in maintaining the structure and function of *AaLOX.*

Among the *AaLOX* genes, 15 were located on eight chromosomes, and the other five were located on unanchored scaffolds and contigs. Therefore, the distribution of *AaLOX* on the chromosomes is uneven. Cis-acting element analysis showed that the majority of cis-acting elements linked to light response were discovered in *AaLOX* genes. Therefore, the *AaLOX* gene might be influenced by the light-induced proteins. Hao [39] found that the increase in artemisinin biosynthesis by JA is dependent on light. However, the relationship between the two signal pathways mediated by JA and light remains unclear. Many cis-acting elements related to biotic and abiotic stress was discovered, such as aphid stress and salt stress [40,41]. On the basis of the expression pattern of *LOX* genes under stress, the *AaLOX* genes might have similar functions. In addition, 28 MeJA response elements were found, thus revealing the potential function of *AaLOX* in response to abiotic stresses in *A. annua*.

Analysis of the transcriptional data of different *A. annua* tissues showed that the expression of *AaLOX* gene family differed in HAN1 and LQ9. *AaLOX* genes were widely distributed in plant organs (including roots, stems, leaves, and flowers) and organelles (chloroplasts and cytoplasm), and therefore may have different functions [19]. The *LOX* located in cytoplasm regulates tuber development and lateral root formation in *A. thaliana* [42], and the *LOX* located in chloroplast plays a role in the ripening and senescence of tomato fruit [43]. JA is an important plant hormone involved in the regulation of plant growth and development. *LOX* is a critical first step in the JA synthesis pathway, and its enzyme activity is positively correlated with JA [44]. qRT-PCR results showed that the expression of *AaLOX5* in the stems of HAN1 and LQ9 was opposite to the transcriptomic data. This difference might have occurred because we collected the same strains from different locations in the same field. *LOX* is a key gene in the JA synthesis pathway, and hormone synthesis is influenced by the environment. The differences in gene expression may be caused by different microenvironments at different locations. Another experiment will be designed to test the influence of microenvironments on gene expression. Although the relative expression of *AaLOX5* and *AaLOX17* in leaves and flowers was relatively high, the relationship between their expression and enzyme activity remains unknown, and the specific *AaLOX* involved in the JA synthesis pathway of *A. annua* needs further verification. In addition, JA treatment increases artemisinin accumulation by simultaneously activating the expression of biosynthetic genes and promoting glandular trichome formation [45]. The synthesis mechanism of artemisinin in *A. annua* is complex and can be affected by multiple regulatory pathways. Further study of the relationship among *AaLOX*, JA, and artemisinin synthesis must be conducted.

## 4. Materials and Methods

### 4.1. Plant Materials and Genome Sequences Acquisition

*A. annua* strains HAN1 and LQ9 were provided by the *A.annua* breeding group in the experimental field of Institute of Chinese Materia Medica, China Academy of Chinese Medical Sciences. The root, stem, leaf and flower tissues of HAN1 and LQ9 were taken and stored in a refrigerator at -80 °C after rapid cooling with liquid nitrogen.

The genomic and transcriptomice data of LQ9 were downloaded from Global Pharmacopoeia Genome Database (GPGD, http://www.gpgenome.com/, accessed on 20 August 2021) [46].

### 4.2. Identification of LOX Genes in A. annua

Pfamscan [47] was used to annotate the protein of *A. annua*, and the Pfam annotation file was obtained. PF00305 was the Pfam number of *LOX* gene retrieved from PFAM database and was used to obtain the *LOX* gene IDs. The genomic data of *A. annua* were uploaded to Apollo [48] and manually corrected according to the *LOX* gene IDs [49]. The corrected CDS and protein sequences of *LOX* were downloaded from Apollo. Potential *LOX* genes were identified using BLASTP and TBLASTN (query length coverage 50% and sequence identification 80%). Finally, the corrected gene was submitted to Pfam protein sequence database (http://pfam.xfam.org/search#tabview=tab0, accessed on 19 February 2020) to identify the members of the *LOX* family, which were named *AaLOX*. In addition, the physical and chemical properties of *AaLOX* protein, such as molecular weight and total average hydrophilicity of theoretical isoelectric point, were predicted using the online tool ExPASy (http://web.expasy.org/protparam/, accessed on 29 January 2022). The online software Cell-PLoc 2.0 (http://www.csbio.sjtu.edu.cn/bioinf/Cell-PLoc-2/, accessed on 25 February 2021) was employed for the subcellular localization and analysis of *AaLOX* proteins.

### 4.3. Chromosome Location Analysis

The whole genome annotation file of *A. annua* and the information on *AaLOX* gene family were imported into TBtools software [50] to determine the chromosome location and draw the corresponding chromosome physical location map.

### 4.4. Selective Pressure Analysis

The *AaLOX* genes database was established by using makeblastdb command, and the *AaLOX* nucleic acid sequences were compared by BLASTN. The ratio of gene Ka (non-synonymous substitution)/Ks (synonymous substitution) was calculated using the Ka/Ks Calculator tool of software TBtools [51] to detect selective pressure on the *AaLOX* gene family. A positive selection effect occurs when Ka/Ks > 1, and a purified selection response occurs when Ka/Ks < 1.

### 4.5. Multiple Sequence Alignment and Evolutionary Analysis

Multiple sequence alignment analysis was carried out by using the MUSCLE tool of MEGA X software [52], and the phylogenetic tree of *AtLOX*-*AaLOX* genes was constructed by the adjacency method (neighbor-joining, NJ). The corresponding *AtLOX* sequences were obtained from the TAIR database (http://www.arabidopsis.org, accessed on 13 April 2021). The number of bootstrap repeats of the check parameter was set to 1000. The phylogenetic tree was visually modified using online software EvolView (https://www.evolgenius, accessed on 17 July 2021).

### 4.6. Gene Structure and Conservative Motif Analysis

The online software GSDS tool (http://gsds.gao-lab.org/index.php, accessed on 13 April 2021) was used to obtain the exon-intron structure of the *AaLOX* protein sequences. The conservative domain of *AaLOX* family protein was predicted and analyzed with the online tool meme (http://meme-suite.org/tools/meme, accessed on 14 April 2021). Motif number was set to 10, and the rest of the parameters were set to default [53]. TBtools was used to extract and visualize the location information of *AaLOX*. In combination with the phylogenetic tree of the *AaLOX* proteins, the gene structure and motif were visually analyzed with TBtools.

### 4.7. Secondary Structure and Tertiary Structure Prediction of AaLOX Proteins

Online websites SOPMA (https://npsa-prabi.ibcp.fr/cgi-bin/secpred_sopma.pl, accessed on 20 November 2021) and SWISS-MODEL (https://swissmodel.expasy.org/interactive/RZm3Df/models/, accessed on 4 December 2021) were used to predict the secondary and tertiary structure of proteins.

### 4.8. Analysis of the Promoter Regions of A. annua

The upstream 2000 bp of transcriptional initiation of family members was defined as the promoter region of *AaLOX* genes for analysis. The elements in the promoter region were predicted using PlantCARE (http://bioinformatics.psb.ugent.be/webtools/plantcare/html/, accessed on 6 September 2021) online website and visualized with Tbtools [54].

### 4.9. GO Enrichment Analysis of AaLOX Genes

For GO functional enrichment analysis, the genome of *A. annua* was annotated by blast2GO.

### 4.10. Expression Analysis of AaLOX Genes

The expression data of different strains and tissues were processed by log (TPM+1), and the expression heat map was drawn using the pheatmap package of R. Total RNA for qRT-PCR was extracted with Aidlab RNA extraction kit (Aidlab, Beijing, China). Samples were obtained from the roots, stems, leaves, and flowers of HAN1 and LQ9 plants and frozen at −80 °C. A Nanodrop-2000 spectrophotometer (Thermo Fisher Scientific, Waltham, MA, USA) was used to determine RNA concentration, purity, and integrity, followed by 1% agarose gel electrophoresis. Total RNA was transformed into cDNA using the FastKing cDNA Synthesis Kit KP118 (Tiangen, Beijing, China). The qRT-PCR primers are shown in Table S4. In brief, 20 μL of mixture was prepared for each sample containing 10 μL of ChamQ Universal SYBR qPCR Master Mix (Vazyme, Nanjing, China), 1 μL of synthesized cDNA product, 0.4 μL of each primer, and 8.2 μL of ddH20. The mixture was subjected to qRT-PCR by SLAN-96S automatic fluorescent quantitative PCR (Hongshi, Shanghai, China) with the following reaction protocols: a denaturation cycle at 95 °C for 30 s, followed by 40 cycles of 95 °C for 10 s and 60 °C for 15 s. Three biological replicates were used for each gene. The relative gene expression level was analyzed using the 2^−ΔΔCT^ method [55].

### 4.11. Artemisinin Content in A. annua Leaves

In brief, 100mg of fresh HAN1 and LQ9 leaves were ground into homogenate with 1 mL of (80% methanol + 0.1% formic acid) extract and then transferred to a volumetric flask to a final volume of 1mL. The flask was kept in an ultrasonic bath for 20 min and then centrifuged at 12,000× *g* for 10 min. Afterward, 800 μL of supernatant was obtained and placed in the injection bottle, stored at −20 °C, and diluted by 100× for further testing.

Ultra-performance liquid chromatography/tandem mass spectrometry (UPLC-MS/MS) with ESI source was used to determine and quantify artemisinin. The chromatographic column was BEH C18 (130A, 1.7 µm, 1 mm × 100 mm, 1/pkg, Waters, made in Ireland). The mobile phase was composed of water (A, 0.1% formic acid) in acetonitrile (B, 0.1% formic acid and 5 mM ammonium formate in 95% acetonitrile), and the gradient program was 0–1.00 min 40% A and 60% B, 1.01–2.50 min 20% A and 80% B, 2.51–2.60 min 5% A and 95% B, and finally 2.61–4.00 40% A and 60% B. The flow rate of the mobile phase was 0.20 mL/min, the column temperature was set to 35 °C, and the injection volume was 1 μL. The parent and daughter ions were 283.2 and 247.1, respectively, and the collision energy was 8 V.

### 4.12. JA Content in A. annua Leaves

In brief, 200mg of fresh HAN1 and LQ9 leaves were ground into homogenate with 1 mL of (methanol + 0.1% formic acid) extract and then transferred to a volumetric flask in a final volume of 1mL. The volumetric flask was kept in an ultrasonic bath for 20 min, then frozen in the refrigerator at -20 °C for 1 h and centrifuged at 10,000× *g* for15 min. Afterward, 800 μL of supernatant was obtained, concentrated to dry with nitrogen blower, re-dissolved in 150 μL initial mobile phase, stored in refrigerator at −20 °C for further testing.

UPLC-MS/MS with ESI source was used to determine and quantify JA. The chromatographic column was BEH C18 (130A, 1.7 µm, 1 mm × 100 mm, 1/pkg, Waters, made in Ireland). The mobile phase was composed of water (A, 0.1% formic acid) in methanol (B, 0.1% formic acid in methanol), and the gradient program was 0–2.00 min 90% A and 10% B, 2.01–4.00 min, 45% A and 65% B, and finally 4.01–5.00 min 90% A and 10% B. The flow rate of the mobile phase was 0.40 mL/min, and the column temperature was set to 40 °C. The injection volume was 1 μL. The parent and daughter ions were 209 and 60, respectively, and the collision energy was 20 V.

## 5. Conclusions

This study identified the *AaLOX* gene family in *A. annua* at the chromosome level and analyzed their structures, conservative structures, evolutionary relationships, and GO enrichment. Expression patterns and qRT-PCR indicated that *AaLOX* genes were expressed differently in different tissues. The precise functions of *AaLOX* proteins remain unknown and thus require further investigations. This work provides a basis for future comprehensive studies on the functional analysis of *AaLOX* proteins. The relationship between the JA synthesis pathway and *AaLOX* would be of great interest to experimental design and should be considered.

## Data Availability

The genome sequences of plant species and RNA-seq data used in manuscript were downloaded from the GPGD website (http://www.gpgenome.com/, accessed on 20 August 2021).

## Supplementary Materials

The following are available online at https://www.mdpi.com/article/10.3390/plants11050655/s1, Figure S1: The tertiary structure of 17 proteins of *AaLOX,* Table S1: The gene family information of *LOX* in *A. annua*, Table S2: Divergence between paralogous *LOX* gene pairs in *A. annua*, Table S3: Secondary structure analysis of *AaLOX* proteins. Table S4: *AaLOX5* and *AaLOX17* primer sequences.
