# Supplementary material for: Genome-Wide Identification, Characterization and Expression Analysis of Lipoxygenase Gene Family in Artemisia annua L
Source: Plants (Basel). 2022 Feb 28;11(5):655. doi: 10.3390/plants11050655 (PMC8912875; doi:10.3390/plants11050655)
Supplement: Supplementary file 1 [file plants-11-00655-s001.zip › Table S1 The gene family information of LOX in A. annua.pdf]

Table S1. The gene family information of *LOX* in *A. annua.*

Gene name	Start	End	Amino acids	MW(Da)	PI	GRAVY	Instability index	subcellular localization
AaLOX1	2585575	2591359	942	107314.1	6.89	-0.452	41.96	Chloroplast. Cytoplasm.
AaLOX2	6877551	6882426	916	103246.9	6.42	-0.349	42.86	chloroplast
AaLOX3	854174	859331	855	97105.7	5.88	-0.38	42.03	Chloroplast. Cytoplasm.
AaLOX4	44677560	44680589	532	60617.39	5.78	-0.257	48.83	Cytoplasm
AaLOX5	76879119	76886931	901	102269.6	5.52	-0.417	42.45	Chloroplast. Cytoplasm.
AaLOX6	63906308	63918611	924	104069.1	5.65	-0.359	41.32	chloroplast
AaLOX7	11748184	11755721	868	98723.29	5.78	-0.433	39.21	chloroplast
AaLOX8	88227373	88231725	905	102497.7	6.68	-0.423	40.05	Chloroplast. Cytoplasm.
AaLOX9	767964	76815047	1217	139133	6.52	-0.395	46.1	chloroplast
AaLOX10	13058884	13069498	871	99650.25	5.51	-0.407	42.1	Chloroplast. Cytoplasm.

AaLOX11	63804505	63812481	924	103970.1	5.65	-0.369	42.65	chloroplast
AaLOX12	880807	891175	887	100541.5	5.5	-0.326	38.68	Chloroplast. Cytoplasm.
AaLOX13	925787	933063	867	99423.62	5.83	-0.505	48.53	Cytoplasm
AaLOX14	76846023	76852677	902	100977.5	5.59	-0.33	36.68	chloroplast
AaLOX15	23	4318	738	83760.86	5.22	-0.397	38.32	chloroplast
AaLOX16	13073867	13087546	868	99089.76	5.73	-0.424	39.77	Chloroplast. Cytoplasm.
AaLOX17	63778878	63788962	853	96495.65	5.25	-0.378	40.64	chloroplast
AaLOX18	13044457	13058394	882	100331.9	5.28	-0.379	43.21	Cytoplasm
AaLOX19	29658	30051	130	15093.37	5.74	-0.504	32.46	Chloroplast. Cytoplasm.
AaLOX20	76859253	76864081	899	101320.9	5.71	-0.381	39.27	chloroplast
