# Supplementary material for: Genome-Wide Identification, Characterization and Expression Analysis of Lipoxygenase Gene Family in Artemisia annua L
Source: Plants (Basel). 2022 Feb 28;11(5):655. doi: 10.3390/plants11050655 (PMC8912875; doi:10.3390/plants11050655)
Supplement: Supplementary file 1 [file plants-11-00655-s001.zip › Table S2 Divergence between paralogous LOX gene pairs in A.pdf]

Table S2. Divergence between paralogous *LOX* gene pairs in *A. annua.*

gene1	gene2	Ka	Ks	Ka/Ks
AaLOX2	AaLOX3	0.026	0.173	0.148
AaLOX5	AaLOX9	0.107	0.617	0.174
AaLOX7	AaLOX8	0.140	0.740	0.189
AaLOX11	AaLOX19	0.001	0.010	0.061
AaLOX16	AaLOX17	0.066	0.435	0.152
AaLOX18	AaLOX20	0.102	0.147	0.696
