# Supplementary material for: Genome-Wide Identification, Characterization and Expression Analysis of Lipoxygenase Gene Family in Artemisia annua L
Source: Plants (Basel). 2022 Feb 28;11(5):655. doi: 10.3390/plants11050655 (PMC8912875; doi:10.3390/plants11050655)
Supplement: Supplementary file 1 [file plants-11-00655-s001.zip › Table S3 Secondary structure analysis of AaLOX proteins.pdf]

Table S3. Secondary structure analysis of *AaLOX* proteins.

Gene name	Secondary structure(%)			
	Alpha helix	Beta turn	Random coil	Extended strand
AaLOX1	39.17	4.99	43.21	12.63
AaLOX2	38.54	5.24	42.47	13.76
AaLOX3	37.31	5.5	43.27	13.92
AaLOX4	43.8	3.95	41.73	10.53
AaLOX5	36.29	5.22	44.28	14.21
AaLOX6	35.06	5.41	45.24	14.29
AaLOX7	37.9	5.53	43.09	13.48
AaLOX8	40.55	4.64	42.32	12.49
AaLOX9	36.15	5.26	44.04	14.54
AaLOX10	37.54	5.17	44	13.2
AaLOX11	35.61	4.55	46	13.85
AaLOX12	36.46	5.64	43.57	14.33
AaLOX13	38.64	5.54	42.21	13.61
AaLOX14	37.36	4.88	45.5	12.2
AaLOX15	38.08	5.28	43.63	13.01
AaLOX16	37.67	5.99	42.86	13.48
AaLOX17	0.3575	4.98	44.53	14.73
AaLOX18	37.19	5.67	43.54	13.61
AaLOX19	46.15	3.85	43.85	6.15
AaLOX20	38.38	5.01	43.6	13.01
