# Supplementary material for: Genome-Wide Identification, Characterization and Expression Analysis of Lipoxygenase Gene Family in Artemisia annua L
Source: Plants (Basel). 2022 Feb 28;11(5):655. doi: 10.3390/plants11050655 (PMC8912875; doi:10.3390/plants11050655)
Supplement: Supplementary file 1 [file plants-11-00655-s001.zip › Table S4 AaLOX5 and AaLOX17 primer sequences.pdf]

Table S4. *AaLOX5* and *AaLOX17* primer sequences.

Gene ID	Forward primer (5′ to 3′)	Reverse primer (5′ to 3′)
AaLOX5	GAGCGACAATCATTTGACAGG	ATGCCACCAAGCACAGGAC
AaLOX17	GGTTCAATCGGGGCAGTT	TGGGGAGTCCTTCAATAA
GAPDH	TCCTAGCAAGGATGCTCCCA	AGGAGCAAGGCAGTTGGTTG
