# Supplementary figures and images for: Genome-Wide Identification, Characterization and Expression Analysis of Lipoxygenase Gene Family in Artemisia annua L
Source: Plants (Basel). 2022 Feb 28;11(5):655. doi: 10.3390/plants11050655 (PMC8912875; doi:10.3390/plants11050655)

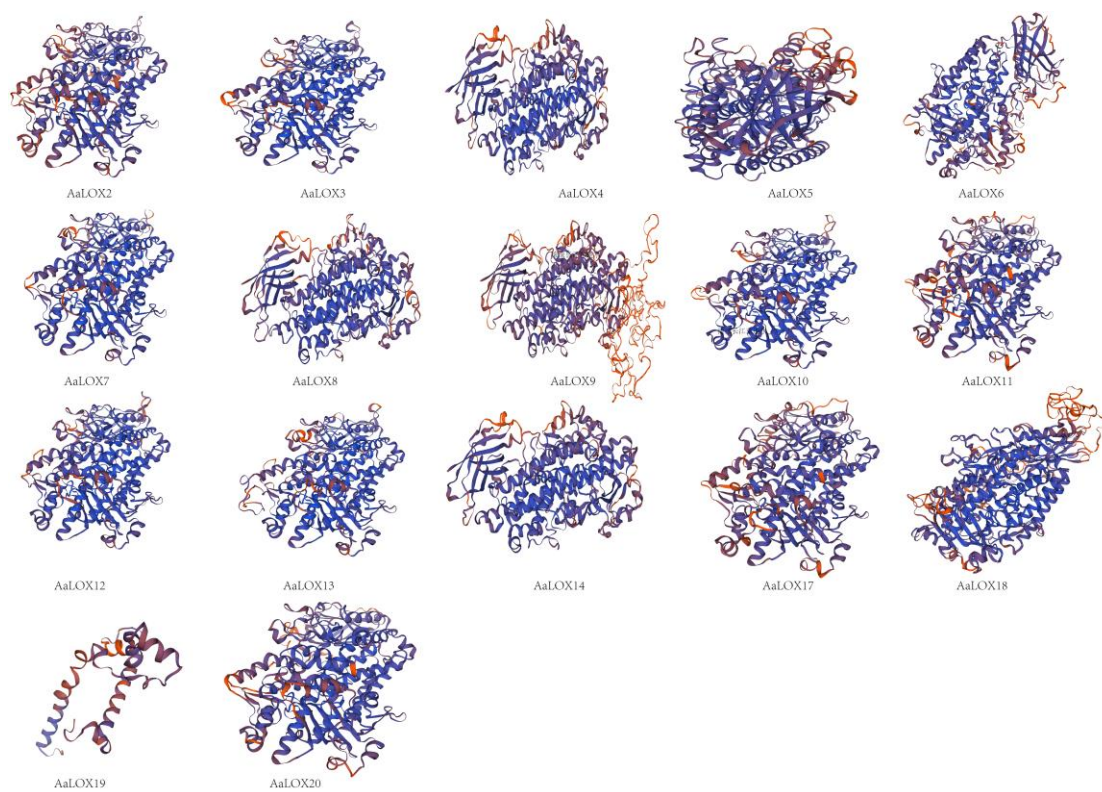

Figure S1. The tertiary structure of 17 proteins of *AaLOX*.

Supplement: Supplementary file 1 [file plants-11-00655-s001.zip › Figure S1 The tertiary structure of 17 proteins of AaLOX.pdf]
